# A High-Voltage Short-Burst Electroacoustic Measurement Method for Piezoelectric Acoustic Logging Transmitters

**DOI:** 10.3390/s26196088

**Published:** 2026-09-25

**Authors:** Kai Zhang, Xinyan Wang, Baohai Tan, Yuanda Su, Lei Liu

**Affiliations:** 1 State Key Laboratory of Deep Oil and Gas, China University of Petroleum (East China), Qingdao 266580, China; tanbaohai@upc.edu.cn (B.T.); syuanda@sina.com (Y.S.); 2 School of Geosciences and Technology, China University of Petroleum (East China), Qingdao 266580, China; z24010048@s.upc.edu.cn; 3 Geological Measurement and Control Technology Research Institute, Sinopec Jingwei Co., Ltd., Qingdao 266071, China; liul28080.osjw@sinopec.com

**Keywords:** acoustic logging, piezoelectric transmitting transducer, high-voltage short-burst excitation, synchronous electroacoustic measurement, dynamic apparent impedance, transmitting voltage response

## Abstract

Piezoelectric acoustic logging transmitters are commonly evaluated using low-voltage small-signal impedance measurements, whereas their practical operation involves high-voltage, high-current, finite-cycle short-burst excitation. This difference makes it difficult to predict the actual loaded electrical input state and directional acoustic response from conventional impedance spectra alone. In this study, a high-voltage short-burst electroacoustic measurement method is developed for piezoelectric acoustic logging transmitters. Unlike conventional small-signal impedance analysis, the proposed method synchronously measures the terminal voltage, terminal current, and 1 m hydrophone response under the same high-voltage short-burst excitation condition. A three-cycle sinusoidal burst was amplified and applied to a water-loaded PZT-5A tube transducer over an 8–100 kHz frequency sweep. For the finite-cycle non-steady-state waveforms, adaptive time-window extraction and single-frequency projection were used to obtain the dynamic apparent impedance, instantaneous power, single-burst input energy, effective short-burst transmitting voltage response, and hydrophone direct-wave voltage energy. The results show that small-signal impedance spectra measured in air and water can identify modal characteristics and candidate frequency bands, but they cannot directly represent the actual high-voltage short-burst operating state. The single-burst input energy shows a stronger frequency-dependent association with the hydrophone direct-wave voltage energy than the impedance magnitude or current amplitude alone. To further evaluate practical applicability, eight nominally identical acoustic logging transmitters were characterized using both conventional small-signal impedance analysis and the proposed high-voltage short-burst method, and their transmitting performances were independently evaluated in a natural-rock model well. The high-voltage dynamic conductance and, in particular, the single-burst input energy showed substantially stronger correspondence with the received P-wave voltage energy than the conventional small-signal conductance. The proposed method provides a practical measurement basis for evaluating acoustic logging transmitters under realistic short-burst excitation conditions, while the eight-transmitter model-well experiment provides preliminary support for its potential use in transmitter screening.

## 1. Introduction

Piezoelectric transducers can efficiently convert electrical energy into mechanical energy and offer fast response, simple excitation, flexible bandwidth design, and broad engineering applicability [1]. They have therefore been widely used in underwater acoustic detection, nondestructive testing, medical ultrasound, and petroleum exploration and development [2,3,4,5,6]. In multi-element ultrasonic transducer arrays, inter-element coupling and crosstalk can also affect the effective vibration response and array performance, and recent studies have reviewed a range of approaches for reducing these effects [7]. In the process of petroleum exploration, acoustic logging tools are deployed thousands of meters underground, where piezoelectric transducers transmit and receive acoustic signals. Recent developments in acoustic-logging instrumentation have also emphasized integrated and reconfigurable test platforms for instrument development and validation, including FPGA-based comprehensive test benches [8]. These signals are then used to evaluate compressional- and shear-wave velocities, estimate formation porosity, identify lithology, and detect fractures and faults [9]. The performance of the transmitting transducer directly affects the detection depth and resolution of the logging tool. Under actual operating conditions, the transmitting transducer is driven by a high-voltage, high-current, short-pulse electrical signal. The excitation voltage can reach several hundred volts to several kilovolts, while the current can reach several amperes. The excitation waveform is usually a short-cycle sinusoidal burst or a pulse signal [10].

The characterization of piezoelectric transmitters generally involves both electrical and acoustic parameters. The electrical parameters mainly include impedance magnitude, phase, resonance frequency, and equivalent circuit parameters [11]. These quantities are usually measured using an impedance analyzer under low-voltage, continuous small-signal conditions, which provides high accuracy, good repeatability, and convenient operation. Acoustic parameters, including sound pressure, acoustic power, transmitting voltage response (TVR), and directivity, are typically measured in a standard underwater acoustic test environment, where the experimental conditions are more demanding and the test procedure is more complex [12].

Conventional impedance analysis can accurately describe the electrical frequency response of a transducer under linear and small-perturbation conditions, but these test conditions differ markedly from the actual operating state. First, the voltage and current at the transducer terminals vary rapidly with time and do not form a steady-state continuous sinusoidal process. Second, under large-signal excitation, the dielectric loss, mechanical loss, and elastic parameters of piezoelectric ceramics may change with electric field strength and vibration amplitude. As a result, the equivalent impedance, quality factor, and resonance state of the transducer may deviate from those obtained from small-signal measurements. Third, the driving circuit has its own output impedance, bandwidth limitation, and current capacity. The voltage actually applied to the transducer may therefore exhibit amplitude reduction, phase shift, waveform distortion, and trailing oscillations.

In recent years, extensive studies have been carried out on testing methods for high-power piezoelectric materials and ultrasonic transducers [11,13,14,15,16,17]. These studies include high-power impedance analysis systems, large-signal impedance measurement, constant-voltage and constant-current excitation tests, burst or transient response characterization, instantaneous input electrical power measurement, acoustic power testing, and electroacoustic efficiency evaluation [13,15,18,19]. The reported results indicate that, under high-power operating conditions, the impedance, loss, quality factor, and acoustic output of piezoelectric transducers are not fixed quantities. Instead, they are closely related to excitation amplitude, temperature rise, loading boundary, driving method, and operating frequency [17,20,21]. Such large-signal tests are useful for revealing nonlinear loss and energy conversion efficiency under realistic working conditions [14,18]. They also show that low-voltage small-signal impedance testing alone is insufficient for fully evaluating the high-power transmitting performance of a transducer [11,15,21].

However, most existing studies have focused on power ultrasonic transducers, medical ultrasonic transducers, or standard piezoelectric material resonators. Their test methods are usually based on continuous sinusoidal excitation, steady-state frequency sweeping, or long-duration burst excitation, which differ substantially from the short-burst operating mode of transmitting transducers used in acoustic logging. Therefore, it remains necessary to clarify how well the results derived from conventional small-signal impedance analysis can represent the actual operating performance of an acoustic logging transducer under high-voltage short-burst excitation. In addition, the excitation effectiveness of the transducer is jointly affected by the loaded electrical response, input energy, waveform distortion, acoustic radiation, and operating frequency.

Building on existing large-signal and transient characterization approaches, this study develops a high-voltage short-burst electroacoustic testing method specifically for piezoelectric transmitting transducers used in acoustic logging. In the experiment, a three-cycle sinusoidal burst, which is commonly used in engineering applications, is employed as the excitation signal. After high-voltage power amplification, the signal is applied to the transmitting transducer. The voltage and current at the transducer terminals are synchronously recorded, together with the hydrophone response measured at a distance of 1 m in a water-tank environment. By acquiring frequency-sweep data over the 8–100 kHz frequency range at 50 Hz intervals, the dynamic apparent impedance, instantaneous input power, and single-burst input energy are calculated. Meanwhile, based on the direct wave recorded by the hydrophone, acoustic indicators such as acoustic output amplitude, direct-wave voltage energy, and transmitting voltage response are extracted. Comparing these parameters with the small-signal impedance spectrum helps clarify the relationships among low-voltage impedance characteristics, high-voltage electrical input capability, and actual acoustic radiation performance. The proposed method therefore provides more direct experimental evidence for the design, impedance matching, and operating-frequency selection of transmitting transducers in acoustic logging tools. In addition to the frequency-sweep characterization of a representative transducer, eight nominally identical transmitters were further tested to examine the practical screening capability of the method. Their low-voltage small-signal electrical characteristics and high-voltage short-burst electrical characteristics were compared with independently measured formation P-wave responses in a natural-rock model well, thereby providing a preliminary application-oriented evaluation of the proposed method.

Previous large-signal and transient characterization studies have mainly focused on impedance variation, power loss, or electroacoustic efficiency under continuous sinusoidal, steady-state, or relatively long-duration excitation. In contrast, the present study focuses on the finite-cycle high-voltage excitation condition of acoustic logging transmitters and synchronously measures the terminal voltage, terminal current, and hydrophone response during the same short burst. The contribution of the proposed method therefore lies in integrating transient electrical characterization and acoustic-response measurement under realistic short-burst operating conditions, rather than in introducing large-signal impedance measurement itself.

## 2. Measurement Principle and Experimental Setup

The high-voltage short-burst synchronous electroacoustic measurement system was constructed as shown in Figure 1. The system mainly consists of a data acquisition and waveform output module, a high-voltage power amplification module, a synchronous voltage/current measurement module, an anechoic water tank, a hydrophone, and the piezoelectric transducer under test. The core control and acquisition unit is a PXIe-9752B data acquisition system from Beijing Art Technology Development Co., Ltd. (Beijing, China). This acquisition card provides analog signal output and is used to generate the excitation control signal in the experiment. During testing, the acquisition system automatically generates a constant-amplitude three-cycle sinusoidal burst at the prescribed frequency and sends this low-voltage control signal to the power amplifier.

The power amplifier is an NYK5887-L6 unit from Ningbo Yongke Acoustic Technology Co., Ltd. (Ningbo, China), with an excitation bandwidth of 100 Hz–100 kHz. After power amplification, the high-voltage short-burst signal, with a peak voltage of up to approximately 800 V in this experiment, is applied to the piezoelectric transducer under test to drive acoustic radiation in water. The tested transducer is a radially polarized PZT-5A piezoelectric ceramic tube transducer (Baoding Hongsheng Acoustics Electron Apparatus Co., Ltd., Baoding, China). The outer diameter, inner diameter, and height of the ceramic body are 62 mm, 53 mm, and 41 mm, respectively. The ceramic element is waterproofed using a polyurethane-based encapsulation. After encapsulation, the outer diameter, inner diameter, and height are approximately 68 mm, 47 mm, and 58 mm, respectively. The experiment was carried out in a 6m×6m×6m anechoic water tank. The transmitter and hydrophone were horizontally arranged near the center of the tank with a center-to-center separation of 1.00m. The midpoint of the transmitter–hydrophone pair coincided approximately with the tank center, and the minimum distance from either transducer to the tank boundaries was approximately 2.5m. For a water sound speed of approximately 1500m/s, the direct arrival occurred at approximately 0.67ms, whereas the earliest geometrically possible boundary-reflected arrival was estimated to occur at approximately 4.0ms. The direct-wave analysis window was terminated before this reflected arrival. Therefore, only the direct-wave response was used in the subsequent analysis. No manufacturer-specified anechoic frequency range was used as an analysis criterion; reflected arrivals were instead excluded in the time domain by the direct-wave window.

Voltage and current measurement channels were arranged synchronously at the output terminal of the power amplifier. The voltage was measured using a customized 100:1 voltage divider probe. The current was measured using a PKC8030L current probe (Xi’an Puke Electronic Technology Co., Ltd., Xi’an, China), with a measurement bandwidth of up to 50 MHz. The voltage, current, and hydrophone signals were connected to the analog input channels of the data acquisition system and recorded synchronously. Batch data acquisition was performed using an automatic frequency-sweeping procedure. The sweep range was 8–100 kHz, with a frequency step of 50 Hz. Data acquisition was controlled using custom-written software (Version 1.2). Signal processing was performed using Python (Version 3.10; Python Software Foundation, Beaverton, OR, USA), and figures were prepared using Origin (Version 2021; OriginLab Corporation, Northampton, MA, USA).

To ensure the reliability of the test results, the key measurement links were calibrated and verified before the experiment. The voltage divider probe and current probe were first calibrated in amplitude. The phase delay between the voltage and current channels was then calibrated using a non-inductive standard resistor. In addition, the synchronization of the acquisition channels, the input range of the sampling system, and the effective bandwidth were tested to ensure that the three-channel data satisfied the requirements for amplitude accuracy, phase consistency, and time synchronization.

To evaluate the practical applicability of the proposed method for transmitter screening, eight nominally identical PZT-5A tube transmitting transducers, denoted as T01–T08, were tested. The transducers had the same nominal ceramic material, dimensions, polarization direction, and encapsulation structure as the transducer used in the preceding frequency-sweep experiment.

Each transducer was first characterized using the conventional impedance analyzer under low-voltage small-signal conditions. The resonance frequency $f_{r}$, antiresonance frequency $f_{a}$, impedance magnitude $|Z_{\mathrm{SS}}|$, phase angle $\theta_{\mathrm{SS}}$, and conductance $G_{\mathrm{SS}}$ at the prescribed operating frequency were extracted. For the eight-transmitter comparison, the prescribed operating frequency was 21 kHz. The same transducers were then tested using the proposed high-voltage short-burst system under identical excitation conditions. The terminal voltage and current were synchronously recorded to obtain the high-voltage dynamic impedance $|Z_{\mathrm{HV}}|$, phase angle $\theta_{\mathrm{HV}}$, dynamic conductance $G_{\mathrm{HV}}$, and single-burst input energy $E_{\mathrm{burst}}$. The conductance was calculated from the real part of the admittance, Y=1/Z=G+jB. The quantities obtained from the high-voltage short-burst test therefore characterize the loaded operating state of the coupled amplifier–transducer–water system under the specified driving condition, rather than intrinsic properties of the transducer alone.

An independent transmitting-performance experiment was subsequently conducted in a natural-rock model well. During these measurements, only the transmitting transducer was replaced, whereas the transmitting position, receiving transducer, source–receiver spacing, excitation waveform, receiving gain, acquisition parameters, and borehole-fluid condition were kept unchanged. A fixed time window containing the first-arriving formation P-wave was selected from the received waveform. The P-wave amplitude $A_{P}$ and windowed received P-wave voltage energy $E_{P}$ were extracted, with

(1) $$E_{P}=\int_{t_{P1}}^{t_{P2}}x^{2}\left(t\right)\;dt,$$

where x(t) is the received voltage waveform and $\left[t_{P1},t_{P2}\right]$ is the fixed P-wave analysis window. In the present experiment, the fixed P-wave analysis window was set to $\left[t_{P1},t_{P2}\right]=\left[0.55,0.85\right]$ ms relative to the excitation onset, covering the first-arriving formation P-wave while excluding the subsequent wave packets. Because the receiving transducer was not calibrated for absolute acoustic energy, $E_{P}$ represents the received P-wave voltage energy rather than absolute formation-wave acoustic energy, and it was used as a relative indicator of transmitting performance in the model well.

The natural-rock model well consisted of a 10-m-deep, 89-mm-diameter borehole in mudstone with a compressional-wave velocity of approximately 2500 m/s. The borehole was filled with ordinary water. The transmitting transducer was positioned below the receiving transducer with a source–receiver spacing of 1.5 m, and the midpoint of the transmitter–receiver pair was located at approximately 5 m depth. A three-cycle 21 kHz sinusoidal burst was used for excitation. The receiving transducer was a conventional PZT-5A tubular acoustic-logging receiver arranged coaxially with the borehole axis. The received signal was acquired at 2 MS/s with 16-bit resolution and a receiving gain of 21 dB. A 0.5–50 kHz band-pass filter was applied to the received waveform. For each transmitter, the model-well measurement was repeated three times under unchanged experimental conditions, and the mean value was used for the subsequent correlation analysis. The relative standard deviation of the received P-wave voltage energy $E_{P}$ ranged from 2.1% to 5.6% among the eight transmitters, indicating acceptable repeatability of the model-well measurements.

## 3. Signal Processing and Parameter Extraction

### 3.1. Dynamic Apparent Impedance

Because a three-cycle burst does not establish a steady-state sinusoidal process, the impedance estimated from the measured voltage and current components is referred to here as the dynamic apparent impedance under short-burst excitation. The measured terminal voltage and current were jointly affected by the power amplifier, the dynamic impedance of the transducer, and the water load, and therefore often exhibited amplitude distortion, trailing oscillations, and phase variations. Inspired by endpoint-detection and adaptive processing methods for nonstationary acoustic transients, an adaptive electrical input window was introduced to identify the effective excitation interval for phasor extraction and energy integration, thereby reducing the influence of residual oscillations and inactive waveform segments [22,23].

During data processing, the DC baselines of the voltage and current were first calculated from the pre-excitation silent segment and subtracted from the raw waveforms, yielding the baseline-corrected voltage v(t) and current i(t). The Hilbert envelopes of the voltage and current waveforms, $A_{v}\left(t\right)$ and $A_{i}\left(t\right)$, were then calculated. After normalization, the two envelopes were combined to construct the joint electrical activity envelope:

(2) $$A_{e}\left(t\right)=\max\left(\frac{A_{v}\left(t\right)}{\max A_{v}\left(t\right)},\frac{A_{i}\left(t\right)}{\max A_{i}\left(t\right)}\right).$$

Within the search interval after excitation, the main peak position of the joint envelope was first determined. Starting from this peak position, the algorithm then searched backward and forward. When the joint envelope continuously decreased below the threshold, the start time $t_{1}$ and end time $t_{2}$ of the adaptive electrical input window were determined, respectively. The threshold was defined as the larger value between 10% of the peak value of the joint envelope and the noise threshold, namely

(3) $$A_{\mathrm{th}}=\max\left(0.10A_{\mathrm{peak}},\mu_{\mathrm{noise}}+4\sigma_{\mathrm{noise}}\right),$$

where $A_{\mathrm{peak}}$ is the peak value of the joint electrical activity envelope, and $\mu_{\mathrm{noise}}$ and $\sigma_{\mathrm{noise}}$ are the mean and standard deviation of the envelope noise in the pre-excitation silent segment, respectively.

The fundamental complex component at the current excitation frequency was extracted within this window using a single-frequency complex projection. For a discrete signal x[n] containing *N* samples in the selected window, the complex fundamental component was calculated as

(4) $${\tilde{X}}_{1}\left(f\right)=\frac{2}{NC_{w}}\sum_{n=0}^{N-1}w\left[n\right]x\left[n\right]\exp\left(-j2\pi ft_{n}\right),$$

where $t_{n}$ is the actual sampling time of the *n*th sample, w[n] is the Hann window, and $C_{w}$ is its coherent gain, defined as

(5) $$C_{w}=\frac{1}{N}\sum_{n=0}^{N-1}w\left[n\right].$$

The corresponding RMS amplitude and phase were calculated as

(6) $$X_{1,\mathrm{rms}}\left(f\right)=\frac{|{\tilde{X}}_{1}\left(f\right)|}{\sqrt{2}},\;\phi_{x}\left(f\right)=\arg\left[{\tilde{X}}_{1}\left(f\right)\right].$$

The phase was referenced to the common acquisition time base through the actual sampling time $t_{n}$, thereby avoiding an artificial phase shift caused by variations in the starting position of the adaptive window. Applying the same projection to the voltage and current signals yielded ${\tilde{V}}_{1}\left(f\right)$ and ${\tilde{I}}_{1}\left(f\right)$, respectively.

A single-frequency complex projection method was used to calculate the component at the current excitation frequency *f*. The dynamic equivalent complex impedance was defined as the ratio of the voltage fundamental phasor to the current fundamental phasor:

(7) $$Z\left(f\right)=\frac{{\tilde{V}}_{1}\left(f\right)}{{\tilde{I}}_{1}\left(f\right)}.$$

The impedance magnitude was

(8) $$\left|Z\left(f\right)\right|=\frac{V_{1,\mathrm{rms}}\left(f\right)}{I_{1,\mathrm{rms}}\left(f\right)}.$$

The phase angle was defined as the difference between the voltage phase and the current phase:

(9) $$\theta \left(f\right)=∠{\tilde{V}}_{1}\left(f\right)-∠{\tilde{I}}_{1}\left(f\right).$$

Accordingly, the dynamic apparent impedance was decomposed into the resistive and reactive components:

(10) Rs(f)=ReZ(f),

(11) Xs(f)=ImZ(f).

Here, $R_{s}$ and $X_{s}$ represent the equivalent active component and equivalent energy-storage component under short-burst excitation, respectively.

In the present data processing, the sampling rate was 2.0 MS/s. For the voltage and current signals, the adaptive electrical input window was searched from the nominal excitation onset to ten excitation periods after the onset. A window boundary was accepted only when the joint electrical activity envelope remained below the threshold for at least half of one excitation period, and the valid window length was constrained between three and ten periods. For the hydrophone signal, the direct-wave window was searched around the theoretical arrival time at the 1 m source–receiver distance using the same envelope-threshold strategy. Hann windowing was used in the single-frequency projection, and the extracted amplitudes were corrected by the coherent gain of the Hann window. Thus, the reported $V_{1,\mathrm{rms}}$, $I_{1,\mathrm{rms}}$, and $V_{h,\mathrm{rms}}$ denote the RMS amplitudes of the fundamental components in the selected finite-cycle windows.

To independently validate the finite-cycle impedance estimator, a non-inductive precision resistor was measured using the same acquisition and signal-processing procedure as that used for the transducer measurements. Three-cycle sinusoidal bursts were applied at representative frequencies of 10, 18, 40, and 60 kHz, and the impedance obtained from the single-frequency complex projection was compared with the reference impedance independently measured using the impedance analyzer. The estimated impedance magnitude agreed with the reference value within 1.2%, while the residual phase error remained within 0.30°. These results indicate that the finite-window complex projection itself introduces only a small estimation error compared with the differences observed between the small-signal and high-voltage transducer measurements.

### 3.2. Instantaneous Power and Single-Burst Input Energy

Instantaneous power and single-burst input electrical energy were calculated using the same adaptive electrical input window. Within the adaptive electrical input window $\left[t_{1},t_{2}\right]$, the instantaneous power was calculated as

(12) p(t)=v(t)i(t).

The net input energy of a single burst, *E*, was obtained by integrating the instantaneous power over this window:

(13) $$E=\int_{t_{1}}^{t_{2}}v\left(t\right)i\left(t\right)\;dt.$$

The discrete form was

(14) $$E\approx \sum_{n=n_{1}}^{n_{2}}v\left[n\right]i\left[n\right]\Delta t,$$

where Δt is the sampling interval, and $n_{1}$ and $n_{2}$ are the sampling points, respectively. Since the integration uses signed instantaneous power, the portion of electrical energy returned to the excitation source is counted as negative. Therefore, *E* represents the net electrical energy actually input to the transducer during a single short-burst excitation, with the unit of J.

### 3.3. Hydrophone Acoustic Response

The effective acoustic response window of the hydrophone signal was determined using the same envelope-based adaptive thresholding strategy as that used for the voltage and current signals. The only difference is that the search was performed within the theoretical arrival-time range of the direct wave. Within this adaptive direct-wave window, the peak-to-peak voltage was defined as the difference between the maximum and minimum voltages, the direct-wave voltage energy was defined as the time integral of the squared voltage within the window, and the amplitude at the excitation frequency was extracted using a single-frequency Fourier projection method after Hann windowing.

The TVR, expressed in dB re 1μPa/V @ 1m, was used to quantify the sound pressure level generated by the transducer at 1m under unit excitation voltage. In the calculation, the fundamental RMS value $V_{1,\mathrm{rms}}$ of the excitation voltage at the current frequency was first extracted. Meanwhile, the amplitude of the hydrophone receiving signal at the same frequency was extracted and converted into the RMS hydrophone output voltage $V_{h,\mathrm{rms}}$. The hydrophone voltage was then converted into sound pressure according to the calibrated hydrophone sensitivity $M_{h}\left(f\right)$. Finally, the sound pressure was divided by the excitation voltage to obtain the directional sound-pressure response per unit voltage. The calculation formula was

(15) $$\mathrm{TVR}\left(f\right)=20\log_{10}\left(\frac{V_{h,\mathrm{rms}}\left(f\right)}{V_{1,\mathrm{rms}}\left(f\right)}\right)-M_{h}\left(f\right),$$

where $M_{h}\left(f\right)$ is the receiving sensitivity of the hydrophone at frequency *f*, with the unit of dB re 1V/μPa. Because the acoustic response was measured using a single hydrophone at a distance of 1 m, the reported hydrophone-based quantities characterize the sound-pressure response in the measurement direction rather than the total radiated acoustic power of the transmitter. The frequency-dependent hydrophone sensitivity $M_{h}\left(f\right)$ used in Equation (15) was taken from the calibration data supplied for the HBK 8103 hydrophone, with a nominal sensitivity of approximately $-211\;\mathrm{dB}\;\mathrm{re}\;1\;V{/}^{–}\mathrm{Pa}$ and an uncertainty of approximately ±1.0dB over the frequency range considered in this study.

### 3.4. Repeatability and Measurement Uncertainty Evaluation

To evaluate the reliability of the high-voltage short-burst electroacoustic measurement results, the repeatability, reproducibility, phase accuracy, and measurement uncertainty of the testing system were analyzed. The repeatability evaluation included short-term stability under repeated excitation with the same clamping condition and reproducibility after reinstallation and testing on different dates. Four representative frequencies, 10kHz, 18kHz, 40kHz, and 60kHz, were selected to cover the main frequency-response regions of the tested transducer. At each frequency, five consecutive repeated excitations were performed under the same experimental conditions. The fundamental RMS voltage $V_{1,\mathrm{rms}}$, fundamental RMS current $I_{1,\mathrm{rms}}$, single-burst input energy $E_{\mathrm{burst}}$, peak-to-peak voltage of the hydrophone received waveform $V_{h,\mathrm{pp}}$, direct-wave voltage energy $E_{h}$, and effective short-burst transmitting voltage response (TVR) were extracted [24,25].

For any test parameter *x*, the mean value, standard deviation, and relative standard deviation (RSD) of repeated measurements are defined as

(16) $$\bar{x}=\frac{1}{N}\sum_{k=1}^{N}x_{k},$$

(17) $$s_{x}=\sqrt{\frac{1}{N-1}\sum_{k=1}^{N}{\left(x_{k}-\bar{x}\right)}^{2}},$$

(18) $${\mathrm{RSD}}_{x}=\frac{s_{x}}{\bar{x}}\times 100\%,$$

where N=5 is the number of repeated excitations. The RSD was used to characterize the short-term stability of the measurement results at each representative frequency. The repeatability results are summarized in Table 1.

As shown in Table 1, the RSD of the fundamental voltage was 1.21–1.46%, while that of the fundamental current was 2.14–2.87%. The RSD of the single-burst input energy was 2.89–4.76%. The hydrophone-related quantities showed somewhat larger variability, with an RSD of 3.27–4.53% for $V_{h,\mathrm{pp}}$ and 4.78–7.13% for $E_{h}$. The standard deviation of the effective short-burst TVR was 0.26–0.51dB. These results indicate that the electrical measurements provide good short-term repeatability, whereas the acoustic measurements are more sensitive to small variations in the experimental environment and spatial alignment.

To evaluate the influence of reinstallation and measurements performed on different dates, the transmitting transducer and hydrophone were removed and reinstalled while maintaining the nominal source–receiver distance of 1m. The same four representative frequencies were then tested again. This procedure was used to evaluate the influence of small variations in transducer orientation, spatial position, clamping condition, and water-tank environment on the electroacoustic response. For the reinstallation tests, the relative deviation between the mean values obtained from different tests was calculated as

(19) $$\delta_{x}=\frac{x_{j}-x_{\mathrm{ref}}}{x_{\mathrm{ref}}}\times 100\%,$$

where $x_{\mathrm{ref}}$ is the result obtained from the first installation, and $x_{j}$ is the result obtained from the *j*th reinstallation or from a test performed on a different date. The corresponding reproducibility results are listed in Table 2.

The electrical parameters were less affected by reinstallation, whereas the hydrophone response showed larger deviations because of the sensitivity of the received acoustic field to transducer orientation, source–receiver alignment, and water-tank environmental conditions. Nevertheless, the overall trends with frequency remained unchanged in the repeated tests.

#### 3.4.1. Voltage–Current Phase Calibration and Residual Phase Uncertainty

Because the dynamic apparent impedance and the signed instantaneous-power integral depend directly on the phase relationship between terminal voltage and current, the relative phase delay between the two measurement channels was calibrated independently. A non-inductive precision resistor was connected as the load, and the voltage and current channels were recorded using the same DAQ channels, sampling rate, probes, and waveform-processing procedure as used in the transducer experiment. The measured phase offset was converted into an equivalent channel delay and compensated during data processing.

After phase-delay compensation, the residual voltage–current phase difference measured with the non-inductive load remained within ±0.30° over 10–60kHz and within ±0.50° over the full 8–100kHz range.

To evaluate the influence of the remaining phase uncertainty on the single-burst input energy, an additional relative time shift corresponding to the residual phase uncertainty was numerically applied to the measured current waveform, and $E_{\mathrm{burst}}$ was recalculated. The resulting variation in $E_{\mathrm{burst}}$ was less than 0.8% at the four representative frequencies.

This result indicates that, after phase-delay correction, the remaining voltage–current phase error contributes less to the uncertainty of $E_{\mathrm{burst}}$ than the amplitude measurement, repeatability, and adaptive-window selection.

#### 3.4.2. Sensitivity to Adaptive Time-Window Selection

The influence of the adaptive time-window parameters was also evaluated because both the dynamic apparent impedance and $E_{\mathrm{burst}}$ depend on the selected finite-cycle interval. The nominal envelope threshold used in this study was 10% of the peak joint electrical activity envelope. For the sensitivity analysis, the threshold was varied to 8%, 10%, and 12%, while all other processing parameters were kept unchanged. In addition, the permitted maximum window length was varied around the nominal value of ten excitation periods. The noise threshold was also varied from $\mu_{\mathrm{noise}}+3\sigma_{\mathrm{noise}}$ to $\mu_{\mathrm{noise}}+5\sigma_{\mathrm{noise}}$, around the nominal value of $\mu_{\mathrm{noise}}+4\sigma_{\mathrm{noise}}$. The resulting variations were less than 0.5% for $|Z_{\mathrm{HV}}|$ and 0.8% for $E_{\mathrm{burst}}$.

Over the four representative frequencies, variation in the envelope threshold from 8% to 12% resulted in changes of less than 0.6% in $V_{1,\mathrm{rms}}$, 0.9% in $I_{1,\mathrm{rms}}$, 1.4% in $|Z_{\mathrm{HV}}|$, and 1.8% in $E_{\mathrm{burst}}$. Changing the permitted maximum window length from eight to twelve excitation periods produced a maximum variation of approximately 1.5% in $E_{\mathrm{burst}}$.

More importantly, the principal frequency-dependent trends and the locations of the main response bands were unchanged under these processing variations. These results indicate that the main conclusions are not sensitive to small changes in the adaptive-window parameters. The observed variations were included as a window-selection contribution in the uncertainty analysis.

#### 3.4.3. Measurement Uncertainty

The uncertainty budget of the high-voltage short-burst electroacoustic measurement system is summarized in Table 3. The main sources include instrument calibration, DAQ quantization, repeatability, residual voltage–current phase uncertainty, adaptive-window selection, hydrophone sensitivity, and reinstallation reproducibility.

For the fundamental RMS voltage, the relative combined standard uncertainty can be expressed as

(20) $$u_{r}\left(V_{1,\mathrm{rms}}\right)=\sqrt{u_{r,V,\mathrm{cal}}^{2}+u_{r,\mathrm{DAQ}}^{2}+u_{r,V,\mathrm{rep}}^{2}+u_{r,V,\mathrm{win}}^{2}}.$$

The relative combined standard uncertainty of the fundamental RMS current is

(21) $$u_{r}\left(I_{1,\mathrm{rms}}\right)=\sqrt{u_{r,I,\mathrm{cal}}^{2}+u_{r,\mathrm{DAQ}}^{2}+u_{r,I,\mathrm{rep}}^{2}+u_{r,I,\mathrm{win}}^{2}}.$$

For the dynamic apparent impedance obtained from the ratio of the voltage and current phasors, the relative uncertainty of its magnitude can be approximately expressed as

(22) $$u_{r}\left(\right|Z_{\mathrm{HV}}\left|\right)=\sqrt{u_{r}^{2}\left(V_{1,\mathrm{rms}}\right)+u_{r}^{2}\left(I_{1,\mathrm{rms}}\right)}.$$

The resistance and reactance components are given by

(23) $$R_{s,\mathrm{HV}}=|Z_{\mathrm{HV}}|\cos\theta ,\;X_{s,\mathrm{HV}}=\left|Z_{\mathrm{HV}}\right|\sin\theta ,$$

where θ is the voltage–current phase difference. Therefore, the uncertainty of $R_{s,\mathrm{HV}}$ and $X_{s,\mathrm{HV}}$ includes both the magnitude uncertainty of $|Z_{\mathrm{HV}}|$ and the residual phase uncertainty. Using first-order uncertainty propagation,

(24) $$u^{2}\left(R_{s,\mathrm{HV}}\right)={\left[\cos\theta \;u(|Z_{\mathrm{HV}}|)\right]}^{2}+{\left[|Z_{\mathrm{HV}}|\sin\theta \;u\left(\theta \right)\right]}^{2},$$

and

(25) $$u^{2}\left(X_{s,\mathrm{HV}}\right)={\left[\sin\theta \;u(|Z_{\mathrm{HV}}|)\right]}^{2}+{\left[|Z_{\mathrm{HV}}|\cos\theta \;u\left(\theta \right)\right]}^{2}.$$

For the single-burst input energy, the main uncertainty contributions arise from voltage and current measurement, residual phase mismatch, adaptive-window selection, and repeatability. Its relative combined standard uncertainty can be approximately expressed as

(26) $$u_{r}\left(E_{\mathrm{burst}}\right)=\sqrt{u_{r,V,\mathrm{cal}}^{2}+u_{r,I,\mathrm{cal}}^{2}+u_{r,E,\phi }^{2}+u_{r,E,\mathrm{win}}^{2}+u_{r,E,\mathrm{rep}}^{2}+u_{r,t}^{2}},$$

where $u_{r,E,\phi }$ denotes the uncertainty contribution caused by the residual voltage–current phase error, and $u_{r,t}$ is the relative uncertainty associated with the sampling interval. Because the sampling clock uncertainty is much smaller than the amplitude-, phase-, repeatability-, and window-related contributions, $u_{r,t}$ is negligible in the present system. The phase contribution $u_{r,E,\phi }$ was evaluated numerically by shifting the measured current waveform according to the residual phase uncertainty and recalculating the signed energy integral.

The uncertainty of the effective short-burst TVR mainly arises from the hydrophone sensitivity, excitation-voltage measurement, hydrophone-output measurement, source–receiver distance, and repeatability [26]. Since TVR is expressed in logarithmic form, its standard uncertainty can be approximately written as

(27) $$\begin{array}{cc} u(\mathrm{TVR})= & \{u^{2}\left(M_{h}\right)+{\left[\frac{20}{\ln10}u_{r}\left(V_{h,\mathrm{rms}}\right)\right]}^{2}+{\left[\frac{20}{\ln10}u_{r}\left(V_{1,\mathrm{rms}}\right)\right]}^{2}\\ \; & +{\left[\frac{20}{\ln10}u_{r}\left(r\right)\right]}^{2}+u_{\mathrm{rep},\mathrm{dB}}^{2}{\}}^{1/2}, \end{array}$$

where $u(M_{h})$ is the uncertainty of the hydrophone sensitivity in dB, $u_{r}\left(V_{h,\mathrm{rms}}\right)$ and $u_{r}\left(V_{1,\mathrm{rms}}\right)$ are the relative standard uncertainties of the hydrophone output voltage and fundamental RMS excitation voltage, respectively, $u_{r}\left(r\right)$ is the relative standard uncertainty of the source–receiver distance, and $u_{\mathrm{rep},\mathrm{dB}}$ is the standard deviation of TVR obtained from repeated measurements. In the present experiment, the source–receiver distance was r=1.00m, with an experimentally determined standard uncertainty of u(r)=0.01m, corresponding to $u_{r}\left(r\right)=1.0\%$. The relative standard uncertainty assigned to $V_{h,\mathrm{rms}}$ was 7.7%, which was estimated using the same hydrophone-channel uncertainty as that used for $V_{h,\mathrm{pp}}$ and $A_{h}\left(f_{\mathrm{exc}}\right)$. Using $u(M_{h})=1.0\;\mathrm{dB}$, $u_{r}\left(V_{h,\mathrm{rms}}\right)=7.7\%$, $u_{r}\left(V_{1,\mathrm{rms}}\right)=2.3\%$, $u_{r}\left(r\right)=1.0\%$, and $u_{\mathrm{rep},\mathrm{dB}}=0.5\;\mathrm{dB}$ gives

$$\begin{array}{cc} u(\mathrm{TVR}) & =[1.0^{2}+{\left(8.686\times 0.077\right)}^{2}+{\left(8.686\times 0.023\right)}^{2}\\ & \;+{\left(8.686\times 0.010\right)}^{2}+0.5^{2}{]}^{1/2}\approx 1.32\;\mathrm{dB}. \end{array}$$

Therefore, the combined standard uncertainty was reported as approximately 1.3dB, and the corresponding expanded uncertainty was 2.6dB for a coverage factor of k=2.

The estimated combined uncertainties of the main extracted parameters are summarized in Table 4. Here, $u_{c}$ denotes the combined standard uncertainty, and *U* denotes the expanded uncertainty with a coverage factor of k=2.

The electrical parameters exhibit lower uncertainties than the hydrophone-related indicators. The combined standard uncertainties of $V_{1,\mathrm{rms}}$ and $I_{1,\mathrm{rms}}$ are approximately 2.3–2.4%, while that of $E_{\mathrm{burst}}$ is approximately 4.5%. The larger uncertainties of the hydrophone-related parameters are mainly associated with hydrophone sensitivity, source–receiver positioning, transducer reinstallation, direct-wave window selection, and small environmental variations in the water tank. The combined standard uncertainty of the effective short-burst TVR is estimated to be approximately 1.3dB. Overall, the repeatability, phase-calibration, and uncertainty analyses indicate that the proposed measurement system provides sufficient stability for comparing the frequency-dependent electrical input state and acoustic response under the same high-voltage short-burst excitation condition.

## 4. Results and Discussion

### 4.1. Typical High-Voltage Three-Channel Waveforms

Figure 2 shows the typical waveforms and frequency-dependent characteristics of the terminal voltage, terminal current, and hydrophone response under high-voltage short-burst excitation. Although the prescribed excitation signal was a three-cycle sinusoidal burst, the voltage actually applied to the transducer terminals varied significantly with frequency, showing clear amplitude variation, waveform distortion, and post-excitation trailing oscillations. This suggests that the terminal voltage is not determined only by the prescribed driving signal, but is jointly affected by the output characteristics of the power amplifier, the frequency-dependent impedance of the transducer, and the acoustic loading in water. Accordingly, the measured terminal voltage, current, dynamic apparent impedance, and hydrophone response should be interpreted as system-level loaded responses under the present drive chain, rather than as intrinsic transmitter characteristics.

The terminal current also exhibits strong frequency-dependent behavior. Its amplitude, phase relationship with the voltage, and oscillation duration vary markedly at different excitation frequencies, reflecting changes in the dynamic apparent impedance and electromechanical coupling state of the transducer under high-voltage excitation. Correspondingly, the amplitude and duration of the hydrophone response vary markedly with excitation frequency of the main direct-wave packet, indicating that the acoustic radiation of the transducer is also frequency selective.

Figure 2d further summarizes the peak-to-peak values of the excitation voltage, current, and hydrophone received waveform over the tested frequency range. The excitation voltage does not remain constant with frequency, which can be attributed to the non-ideal voltage-source behavior of the high-voltage power amplifier. In practical operation, the amplifier output is limited by its output impedance, current-driving capability, frequency bandwidth, and the load impedance of the transducer. The local recovery of the voltage peak-to-peak value near 40 kHz may be associated with a relatively favorable matching condition among the transducer impedance, structural vibration mode, and amplifier output characteristics in this frequency range. These results demonstrate that the prescribed excitation signal, terminal voltage, terminal current, and acoustic response do not follow a simple one-to-one relationship. Therefore, the transmitting performance of acoustic logging transducers should be evaluated using synchronous electrical and acoustic measurements rather than only low-voltage impedance spectra or a single voltage-amplitude parameter.

### 4.2. Comparison Between Small-Signal Impedance and High-Voltage Dynamic Apparent Impedance

Figure 3 compares the impedance characteristics of the tested PZT-5A tube transducer under different testing conditions. The in-air and in-water results were measured using a conventional impedance analyzer (TH2840B), while HV denotes the dynamic apparent impedance calculated from the voltage and current waveforms. The transducer wiring, fixture, and other testing conditions were kept identical in the three measurements.

Figure 3a shows the variation in the equivalent resistance component $R_{s}$. The in-air result exhibits clear high-*Q* resonance characteristics, with sharp resistance peaks around 16–18kHz and 40–45kHz. In contrast, the in-water peaks are significantly reduced and broadened, and the low-frequency peak is largely suppressed. This can be attributed to the added mass and radiation damping introduced by the water load. The $R_{s,\mathrm{HV}}$ differs more clearly from the small-signal results. Its overall amplitude is lower, and only a broad fluctuation is observed near 40kHz, indicating that the transducer does not reach a steady-state resonance condition during short-burst excitation.

Figure 3b shows the variation in the equivalent reactance component $X_{s}$. The small-signal curves show typical capacitive behavior and rapid changes near the resonance bands, especially under the air-load condition. In comparison, the $X_{s,\mathrm{HV}}$ curve changes more gradually and does not show the abrupt reactance variation observed in the small-signal impedance.

Figure 3c shows the phase difference between voltage and current. Both small-signal results show a clear phase transition near 40kHz, indicating that this frequency band corresponds to the main electromechanical coupling mode of the transducer. The phase transition is sharper in air and more gradual in water, consistent with the reduction in resonance quality factor caused by the water load. The HV phase curve also shows a transition toward a smaller phase difference near the main response band, indicating that the high-voltage short-burst test can still capture the main operating mode of the transducer.

Overall, Figure 3 shows that conventional small-signal impedance testing can identify the basic resonance characteristics of the transducer, but it cannot fully represent the electrical input behavior under actual high-voltage short-burst excitation. The HV dynamic apparent impedance represents the combined effects of high voltage, short-burst excitation, water loading, and the practical driving circuit. Therefore, for acoustic logging transmitting transducers, impedance results should be interpreted together with the measured terminal voltage, terminal current, input energy, and hydrophone response.

### 4.3. Instantaneous Power and Single-Burst Input Electrical Energy

Figure 4 further analyzes the instantaneous power and single-burst input electrical energy of the transducer under high-voltage short-burst excitation. As shown in Figure 4a, the instantaneous power waveform varies markedly with frequency in terms of amplitude, polarity alternation, and duration. In frequency bands where the voltage–current phase difference is large, the positive and negative power components alternate more clearly, indicating that part of the energy is exchanged between the driving system and the transducer rather than being effectively delivered. In contrast, near the main response bands, the instantaneous power amplitude increases and the positive power component becomes more dominant, suggesting more effective electrical energy input. At higher frequencies, the instantaneous power response weakens, which indicates a reduced energy transfer capability under the combined effects of dynamic impedance variation, phase relationship, and limited high-frequency driving performance.

Figure 4b compares the frequency responses of the single-burst input electrical energy, fundamental current amplitude, and dynamic series resistance. The input energy does not simply coincide with either the minimum dynamic resistance or the maximum current amplitude. Instead, higher input energy generally appears in frequency regions where a relatively strong current response and a distinct active impedance component coexist. At some frequencies, a large current mainly reflects reactive energy exchange and does not necessarily lead to high effective energy input. Similarly, the variation in dynamic resistance near the main response band does not alone determine the input energy, because the current amplitude and voltage–current phase relationship also play important roles. These results indicate that the effective electrical input under high-voltage short-burst excitation is jointly controlled by voltage amplitude, current amplitude, phase difference, and dynamic impedance. Therefore, for acoustic logging transmitting transducers, operating-frequency selection and performance evaluation should not rely only on impedance magnitude or current amplitude; instantaneous power and single-burst input electrical energy provide a more direct basis for evaluating the actual electrical input state.

### 4.4. Frequency Response of Hydrophone Acoustic Output

Figure 5 compares the hydrophone response and transmitting voltage response (TVR) of the transducer under high-voltage short-burst excitation. The hydrophone response exhibits clear frequency selectivity. The three indicators reach their maximum values around 15–18kHz, indicating the strongest hydrophone-measured acoustic response in the measurement direction under the present experimental conditions. A secondary response peak appears near 40–45kHz, suggesting that the transducer still has relatively strong acoustic radiation in this band.

The hydrophone response is not determined only by the intrinsic radiation efficiency of the transducer, but also by the actual driving state between the power amplifier and the transducer. A large received signal may result from a higher directional sound-pressure response per unit voltage, or from larger applied voltage, stronger current response, or more effective input energy at that frequency. Thus, the hydrophone response reflects the combined effect of the driving system, transducer, and water load.

Figure 5b further shows the voltage-normalized transmitting characteristics. Relatively strong TVR values are observed near 15–18kHz and 40–45kHz, generally consistent with the enhanced frequency bands in Figure 5a. Combined with Figure 4, frequency bands with larger single-burst input energy usually correspond to stronger direct-wave voltage energy, indicating that effective electrical input is an important basis for the hydrophone-measured directional response. However, the relationship is not strictly linear, because part of the input energy is dissipated through dielectric loss, mechanical loss, encapsulation damping, and local structural vibration.

Overall, Figure 5a reflects the hydrophone-measured directional acoustic response under the present high-voltage driving system, whereas Figure 5b represents the voltage-normalized acoustic response extracted from the short-burst signal, and should therefore be interpreted as an effective short-burst TVR rather than a steady-state TVR. For the tested transducer, the strongest hydrophone-measured acoustic response occurs around 15–18kHz, with a secondary peak near 40–45kHz. Under a given driving circuit and loading condition, the effective operating frequency should be determined by jointly considering the high-voltage electrical input, effective short-burst TVR, and hydrophone response. The strongest hydrophone response near 15–18kHz does not necessarily coincide with the principal small-signal phase transition near 40kHz because the two measurements describe different operating conditions. The small-signal phase transition mainly reflects the electromechanical resonance behavior under low-voltage steady-state excitation, whereas the hydrophone response under three-cycle high-voltage excitation is additionally affected by the actual terminal voltage, current-driving capability, finite-cycle energy transfer, water loading, and radiation efficiency. Consequently, a lower-frequency mode can produce a stronger short-burst directional acoustic response even when the most pronounced small-signal phase transition occurs at a higher frequency.

To further quantify the relationship between the electrical input state and the acoustic output, correlation analysis was performed using the frequency-sweep data. The correlation analysis included 1841 frequency samples over the 8–100 kHz range at 50 Hz intervals. The electrical indicators included the single-burst input energy *E*, the fundamental current amplitude $I_{1}$, and the high-voltage dynamic series resistance $R_{s,\mathrm{HV}}$. The hydrophone response indicators included the peak-to-peak voltage of the received waveform $V_{h,\mathrm{pp}}$, the direct-wave voltage energy $E_{h}$, and the amplitude at the excitation frequency $A_{h}\left(f_{\mathrm{exc}}\right)$. Pearson correlation coefficients were used to quantify the linear frequency-dependent association, whereas Spearman correlation coefficients were calculated to examine the consistency of the frequency-dependent variation trends. Because adjacent frequency points are not statistically independent, these coefficients are interpreted primarily as measures of frequency-dependent association between the electrical and acoustic indicators, rather than as correlations among fully independent observations. The results are summarized in Table 5.

The single-burst input energy *E* shows the strongest overall frequency-dependent association with the hydrophone response indicators. Its Pearson correlation coefficients with $V_{h,\mathrm{pp}}$, $E_{h}$, and $A_{h}\left(f_{\mathrm{exc}}\right)$ are 0.696, 0.676, and 0.655, respectively. The corresponding Spearman correlation coefficients are 0.772, 0.824, and 0.777, indicating that the frequency-dependent variation in *E* is highly consistent with that of the hydrophone output. In comparison, the corresponding association coefficients obtained using the fundamental current amplitude $I_{1}$ are lower, with Pearson coefficients of 0.423–0.447. This suggests that a large current response does not necessarily correspond to strong acoustic radiation, because part of the current may be associated with reactive energy exchange rather than effective energy input. As a robustness check, the correlation analysis was repeated after systematic resampling of the frequency-sweep data at 500 Hz intervals. For example, the Pearson and Spearman correlation coefficients between $E_{\mathrm{burst}}$ and $E_{h}$ changed only from 0.676 and 0.824 to 0.67 and 0.81, respectively. This result indicates that the main frequency-dependent association was not an artifact of the dense sampling of adjacent frequency points.

The dynamic series resistance $R_{s,\mathrm{HV}}$ shows a moderate frequency-dependent association with $V_{h,\mathrm{pp}}$ but much weaker associations with $E_{h}$ and $A_{h}\left(f_{\mathrm{exc}}\right)$. This indicates that the resistive component of the dynamic apparent impedance alone cannot determine the acoustic output level under high-voltage short-burst excitation. In contrast, the single-burst input energy incorporates the combined effects of terminal voltage, terminal current, voltage–current phase relationship, and excitation duration. Therefore, it provides a more direct electrical indicator for evaluating the effective excitation state of the transducer. Nevertheless, the frequency-dependent association between *E* and the hydrophone response is not perfectly linear, because part of the input electrical energy is dissipated through dielectric loss, mechanical loss, encapsulation damping, local structural vibration, and other loss mechanisms before being converted into far-field acoustic radiation.

### 4.5. Application to Transmitter Screening in a Natural-Rock Model Well

Table 6 summarizes the eight-transmitter comparison. The frequency-sweep results above demonstrate the differences between small-signal impedance characteristics and the electrical input state under high-voltage short-burst excitation for a representative transducer. To determine whether these differences are also relevant to practical transmitter screening, the eight nominally identical transducers were further compared using the two electrical characterization approaches and independently evaluated in the natural-rock model well.

The electrical characterization and model-well results are reported in Table 6. Although the eight transducers had nominally identical structures, clear sample-to-sample differences were observed under both small-signal and high-voltage short-burst conditions. More importantly, the relative electrical characteristics obtained using the two measurement methods were not fully consistent. For example, T03 exhibited a relatively high small-signal conductance of 27.8 mS and a resonance frequency close to the prescribed operating frequency, whereas its single-burst input energy and model-well received P-wave voltage energy were only moderate. In contrast, T08 showed only moderate small-signal conductance but exhibited the highest high-voltage dynamic conductance, single-burst input energy, and received P-wave voltage energy.

As shown in Figure 6a, the conventional small-signal conductance $G_{\mathrm{SS}}$ shows only a weak-to-moderate relationship with the independently measured received P-wave voltage energy, with a Pearson correlation coefficient of r=0.383. The data points are relatively scattered, indicating that a relatively high small-signal conductance does not necessarily correspond to strong transmitting performance under practical short-burst excitation.

The relationship becomes substantially stronger when the conductance is determined under the actual high-voltage short-burst condition. Figure 6b shows a strong positive relationship between $G_{\mathrm{HV}}$ and $E_{P}$, with r=0.889. This indicates that the dynamic electrical state measured under practical excitation conditions provides a more representative description of the relative transmitting performance than the conventional small-signal conductance.

Among the investigated electrical indicators, the single-burst input energy shows the strongest correspondence with the model-well response. As shown in Figure 6c, the Pearson correlation coefficient between $E_{\mathrm{burst}}$ and $E_{P}$ reaches r=0.921. Because $E_{\mathrm{burst}}$ incorporates the actual terminal voltage, current, phase relationship, and excitation duration, it provides a more direct measure of the electrical energy delivered to the transducer during one finite-cycle excitation.

Considering the limited sample size (n=8), the 95% confidence intervals of the Pearson correlation coefficients were 0.49–0.98 for $G_{\mathrm{HV}}$ versus $E_{P}$ and 0.62–0.99 for $E_{\mathrm{burst}}$ versus $E_{P}$. The corresponding Spearman rank correlation coefficients were ρ=0.881 and ρ=0.905, respectively. A leave-one-out analysis was also performed by removing each transmitter in turn and recalculating the Pearson correlation coefficients. The resulting coefficients ranged from 0.854 to 0.932 for $G_{\mathrm{HV}}$ versus $E_{P}$ and from 0.894 to 0.940 for $E_{\mathrm{burst}}$ versus $E_{P}$, indicating that the observed relationships were not dominated by any single transmitter.

Figure 6d compares the P-wave energies of the eight transmitters. T08, T04, and T06 exhibit the strongest formation-wave responses, whereas T01 and T05 show the weakest responses. This ranking is not directly reproduced by the conventional small-signal conductance, whereas the high-voltage dynamic conductance and particularly the single-burst input energy show much better consistency with the independently measured model-well performance.

The present screening results should therefore be interpreted as a comparison of transmitters under a fixed electronic drive chain and loading condition. Different power amplifiers, matching networks, or excitation conditions may change the measured system-level indicators and the corresponding operating-frequency response. Because the present validation involved only eight transmitters, these results should be regarded as a preliminary proof-of-concept validation of the proposed screening approach under the present fixed driving and model-well conditions, rather than as a general screening criterion. Further validation using a larger transmitter population is required to establish its broader applicability.

## 5. Conclusions

This study developed a high-voltage short-burst electroacoustic measurement method for piezoelectric transmitting transducers used in acoustic logging. Unlike conventional low-voltage small-signal impedance analysis, the proposed method synchronously measures the terminal voltage, terminal current, and 1 m hydrophone response under the same high-voltage finite-cycle excitation condition. Based on adaptive time-window extraction and single-frequency projection, the dynamic apparent impedance, instantaneous power, single-burst input energy, effective short-burst TVR, and hydrophone direct-wave voltage energy were extracted from non-steady-state short-burst waveforms. The main conclusions are as follows.

First, small-signal impedance measurements in air and water can identify the modal characteristics of the transducer and provide useful candidate frequency bands. The in-air impedance spectrum highlights the intrinsic structural modes, whereas the in-water impedance spectrum reflects the influence of acoustic loading. However, these small-signal results cannot be directly regarded as the actual operating state under high-voltage short-burst excitation. Therefore, impedance analyzer results are more suitable for modal identification and preliminary frequency screening, rather than as the final criterion for operating-frequency selection.

Second, under high-voltage short-burst excitation, the actual terminal voltage and current differ markedly from the prescribed excitation waveform and vary significantly with frequency. This demonstrates that the loaded electrical input state is jointly determined by the output capability of the power amplifier, the dynamic impedance of the transducer, the water load, and the finite-cycle excitation waveform. Therefore, the prescribed driving voltage cannot replace the measured terminal voltage, and performance evaluation based only on voltage amplitude, impedance minimum, or current maximum may lead to biased conclusions.

Third, under the present amplifier–transducer–water-load condition, the strongest hydrophone response of the tested PZT-5A tube transducer occurs around 15–18 kHz, with a secondary acoustic response peak near 40–45 kHz. These frequency bands are not fully consistent with the main features of the small-signal impedance spectra, indicating that the frequency corresponding to the maximum hydrophone-measured directional response should be confirmed by high-voltage electroacoustic measurement rather than inferred only from low-voltage impedance data.

Fourth, the single-burst input energy provides a more direct electrical indicator of the effective excitation state than impedance magnitude or current amplitude alone. Across the frequency sweep, it shows good overall frequency-dependent correspondence with the hydrophone direct-wave voltage energy, because it incorporates the combined effects of terminal voltage, terminal current, phase relationship, and excitation duration. Nevertheless, the relationship between input electrical energy and the hydrophone-measured far-field response is not strictly linear, owing to dielectric loss, mechanical loss, encapsulation damping, local structural vibration, and acoustic radiation efficiency.

Fifth, the effective short-burst TVR and hydrophone direct-wave response provide complementary information. The hydrophone response reflects the directional acoustic response measured at the hydrophone position under the present driving system, whereas the effective short-burst TVR represents the voltage-normalized acoustic response extracted from finite-cycle waveforms. Therefore, the operating frequency of an acoustic logging transmitting transducer should be selected by jointly evaluating the dynamic electrical input, single-burst input energy, waveform distortion, effective short-burst TVR, and hydrophone direct-wave output.

Sixth, the multi-transducer experiment in the natural-rock model well provides preliminary support for the practical applicability of the proposed method. The conventional small-signal conductance showed only limited correspondence with the independently measured received P-wave voltage energy, whereas the high-voltage dynamic conductance showed a substantially stronger relationship. Among the investigated electrical indicators, the single-burst input energy exhibited the strongest correspondence with the received P-wave voltage energy. These preliminary results indicate its potential value for comparative screening of acoustic logging transmitters under realistic finite-cycle excitation. However, validation using a larger transmitter population is required before a general screening criterion can be established.

In summary, the proposed method provides a practical basis for evaluating and screening acoustic logging transmitters under specified high-voltage driving and loading conditions, and for analyzing the interaction among the driving circuit, transducer, and acoustic load. Conventional small-signal impedance analysis remains useful for modal identification and preliminary frequency screening, whereas the proposed method provides direct information on the actual high-voltage loaded electrical state. The multi-transducer model-well experiment provides preliminary evidence of the potential value of single-burst input energy for comparative transmitter evaluation. This study still has several limitations. Temperature was not measured synchronously, and the present validation was restricted to one nominal transducer structure and one principal burst-duration condition. Further work will include synchronous temperature monitoring, transducers with different structures and encapsulation designs, and verification under different burst cycles and driving circuits.

## Figures and Tables

**Figure 1:**
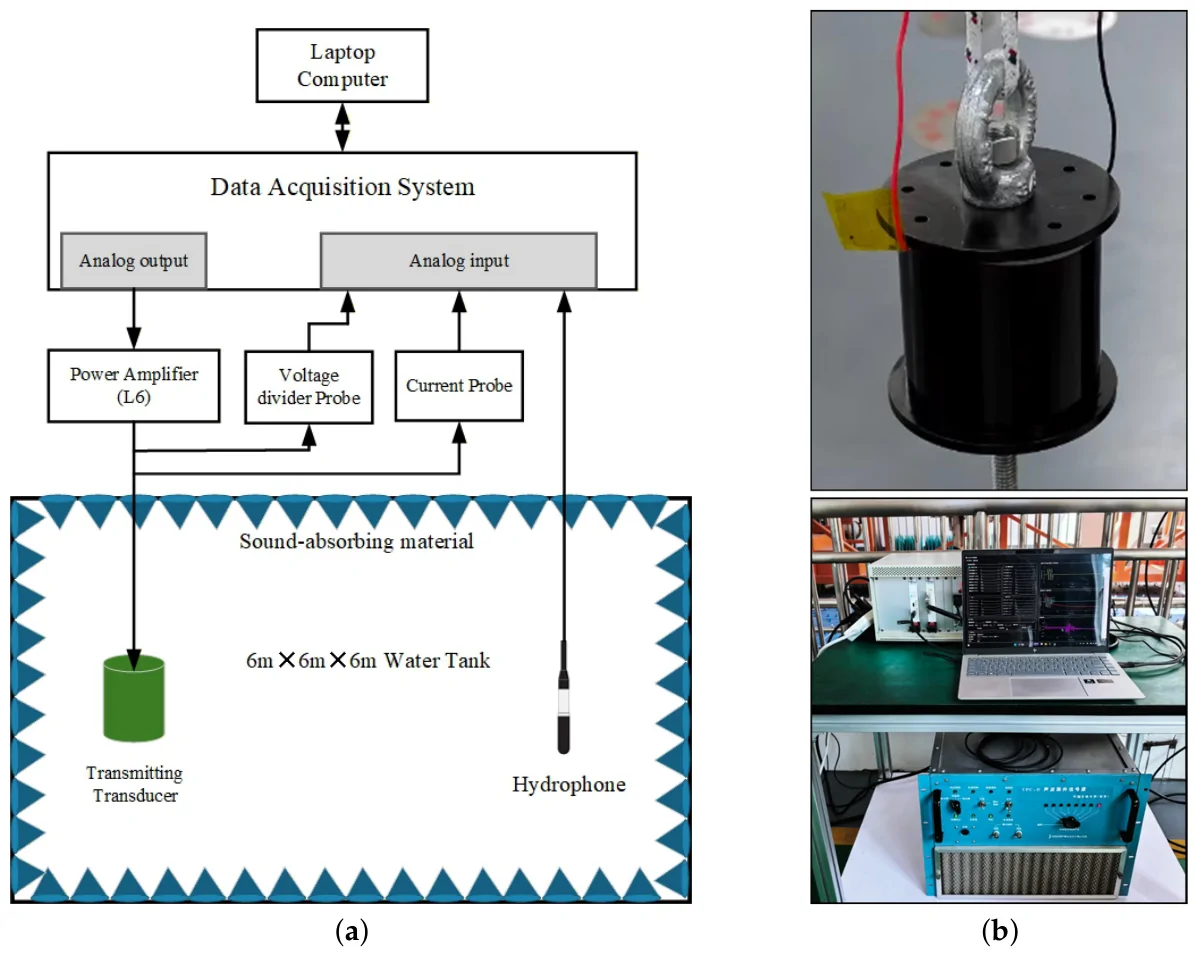
High-voltage short-burst synchronous electroacoustic measurement system. (**a**) Schematic diagram of the testing system; (**b**) photograph of the transmitting transducer, signal generation module, power amplification module, and data acquisition setup.

**Figure 2:**
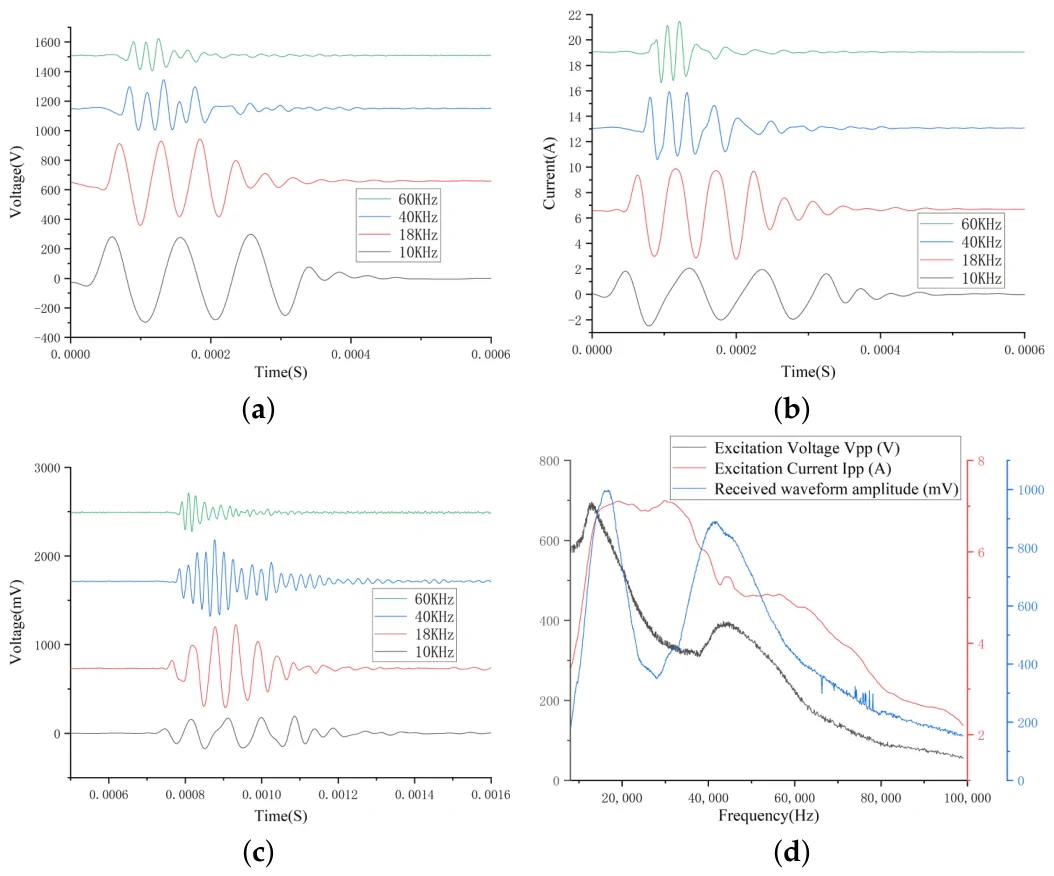
Typical electroacoustic response characteristics of the transducer under high-voltage short-burst excitation. (**a**) Terminal excitation voltage waveforms of the transducer at representative frequencies; (**b**) corresponding current waveforms; (**c**) hydrophone received waveforms at a distance of 1m; (**d**) variations in the peak-to-peak excitation voltage, peak-to-peak current, and hydrophone received waveform amplitude with frequency.

**Figure 3:**
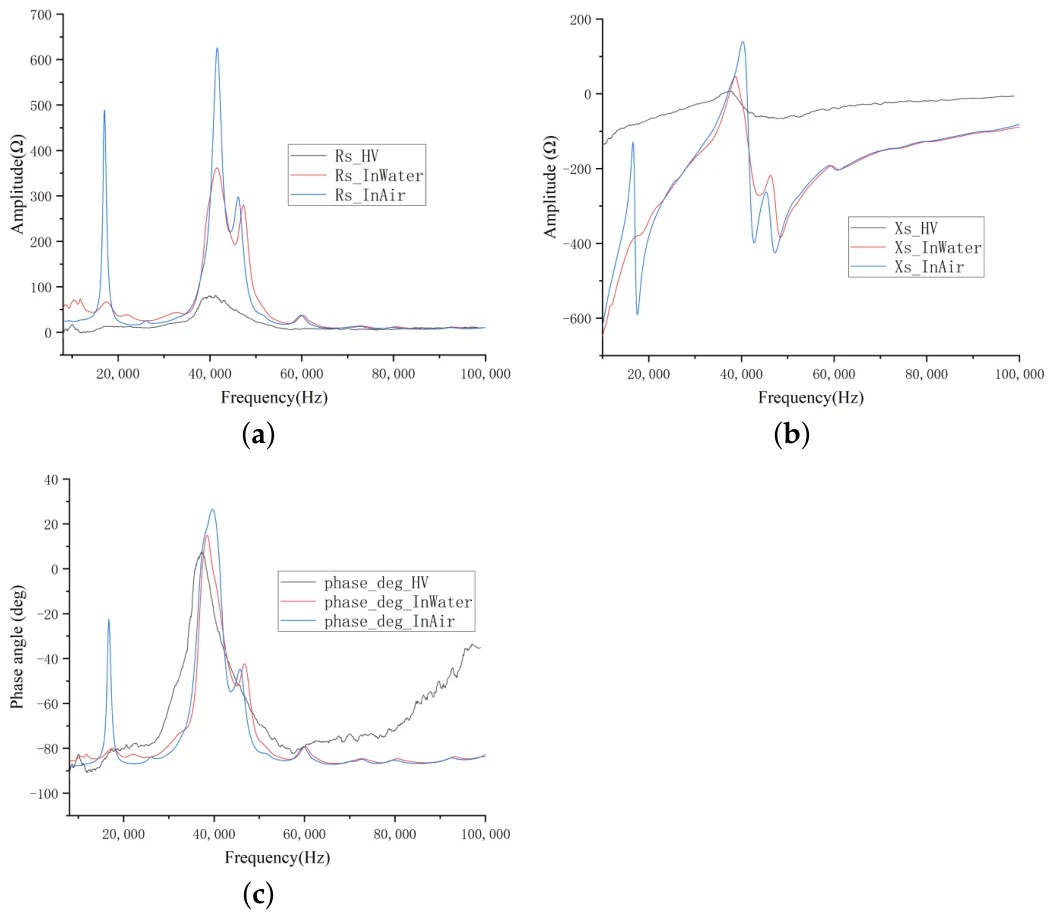
Comparison of small-signal impedance and high-voltage dynamic apparent impedance of the PZT-5A tube transducer. (**a**) Equivalent resistance component $R_{s}$; (**b**) equivalent reactance component $X_{s}$; (**c**) phase difference between voltage and current.

**Figure 4:**
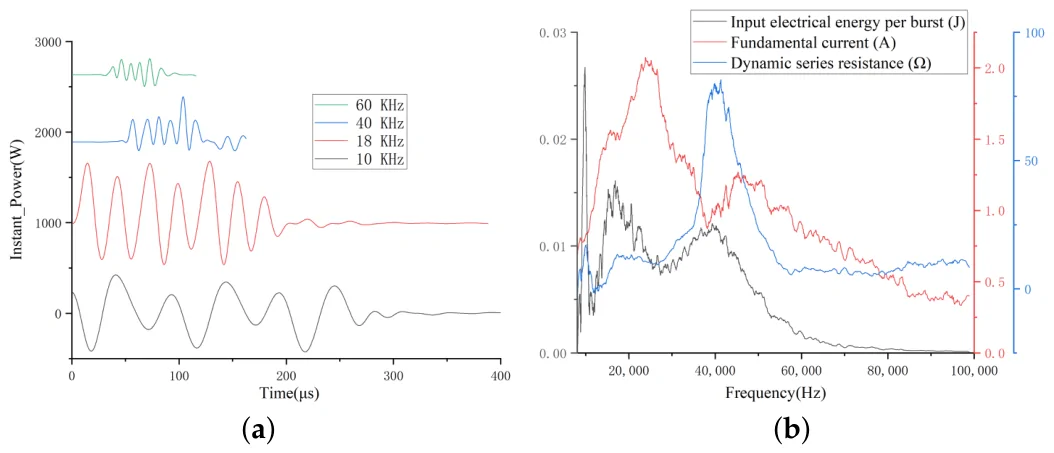
Instantaneous power and single-burst input energy under high-voltage short-burst excitation. (**a**) Instantaneous power waveforms at representative frequencies; (**b**) variations in the single-burst input electrical energy, fundamental current amplitude, and dynamic series resistance with frequency.

**Figure 5:**
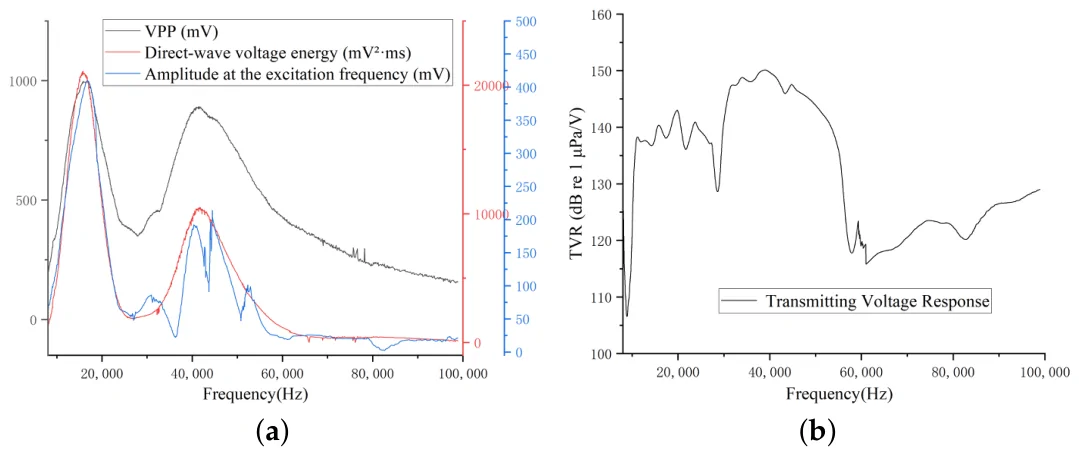
Hydrophone response and transmitting voltage response of the transducer under high-voltage short-burst excitation. (**a**) Variation in the 1 m hydrophone response with excitation frequency. (**b**) Variation in the effective short-burst transmitting voltage response (TVR) calculated from the finite-cycle signal with frequency.

**Figure 6:**
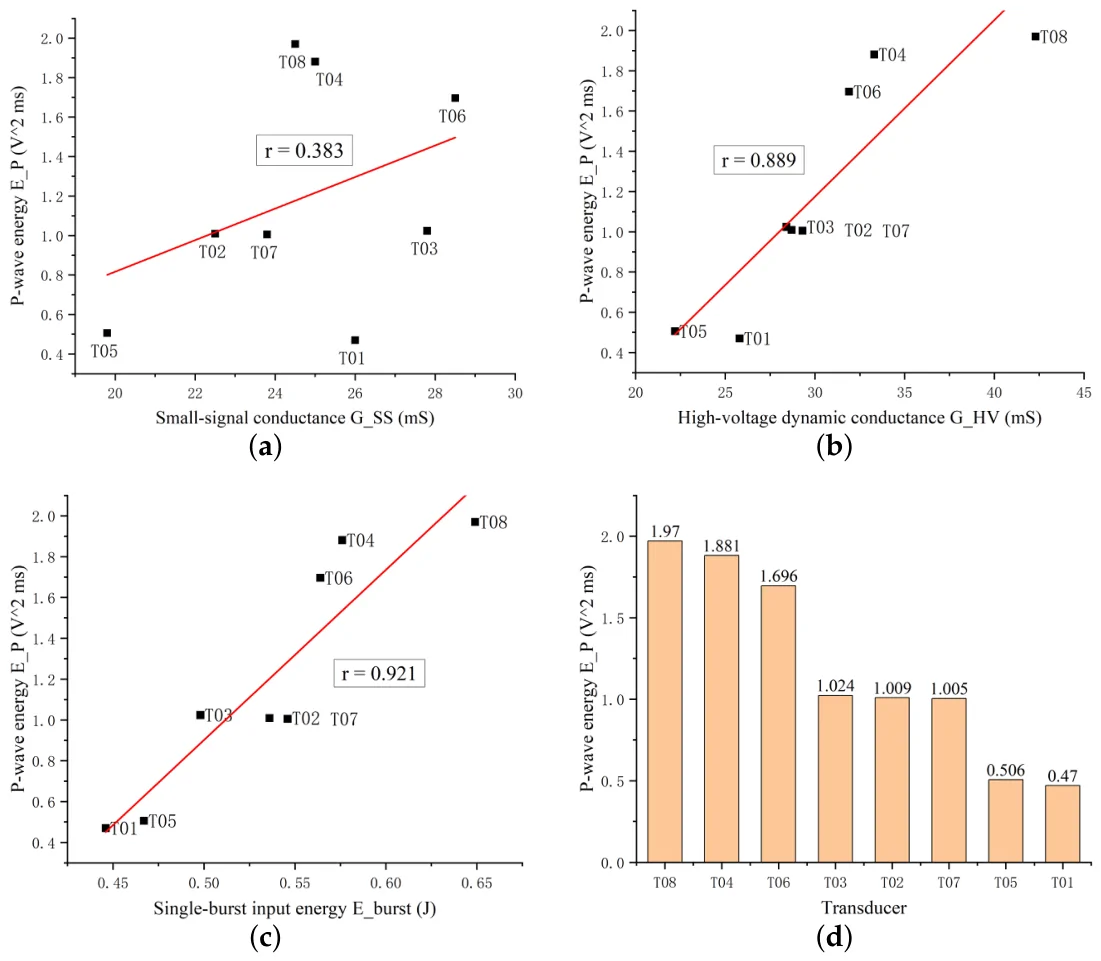
Comparison between electrical indicators and independently measured model-well transmitting performance for eight nominally identical transducers. (**a**) Conventional small-signal conductance $G_{\mathrm{SS}}$ versus received P-wave voltage energy $E_{P}$; (**b**) high-voltage dynamic conductance $G_{\mathrm{HV}}$ versus $E_{P}$; (**c**) single-burst input energy $E_{\mathrm{burst}}$ versus $E_{P}$; (**d**) measured P-wave energies of the eight transmitting transducers in the natural-rock model well.

**Table 1:** Repeatability of the measured electrical and acoustic parameters at representative frequencies.

Frequency (kHz)	$V_{1,\mathrm{rms}}$ RSD (%)	$I_{1,\mathrm{rms}}$ RSD (%)	$E_{\mathrm{burst}}$ RSD (%)	$V_{h,\mathrm{pp}}$ RSD (%)	$E_{h}$ RSD (%)	TVR SD (dB)
10	1.33	2.87	4.76	4.53	6.80	0.51
18	1.21	2.14	2.89	3.27	5.42	0.26
40	1.25	2.61	3.11	3.40	4.78	0.43
60	1.46	2.70	4.24	3.91	7.13	0.45

**Table 2:** Reproducibility of the measurement results after reinstallation and on different test days.

Frequency (kHz)	Relative Deviation (%)				TVR Deviation (dB)
	$V_{1,\mathrm{rms}}$	$I_{1,\mathrm{rms}}$	$E_{\mathrm{burst}}$	$V_{h,\mathrm{pp}}$	
10	2.71	3.48	6.43	8.21	0.57
18	2.69	3.25	5.98	6.47	0.43
40	2.27	3.71	5.40	7.28	0.52
60	3.25	3.45	6.61	9.10	1.23

**Table 3:** Uncertainty budget of the high-voltage short-burst electroacoustic measurement system.

Source of Uncertainty	Instrument/Source	Affected Parameter	Evaluation Type	Value Used in Calculation
Voltage-channel amplitude accuracy	PXIe-9752B DAQ + 100:1 voltage divider	$V_{1,\mathrm{rms}}$, *Z*, $E_{\mathrm{burst}}$, TVR	B	±1.0% relative accuracy
DAQ quantization	PXIe-9752B DAQ	$V_{1}$, $I_{1}$, $V_{h}$	B	18-bit resolution; LSB=76.3μV; $u_{q}=22.0\;\mu V$
Current-probe calibration	PRBTEK PKC8030L	$I_{1,\mathrm{rms}}$, *Z*, $E_{\mathrm{burst}}$	B	±1.0% relative accuracy
Residual voltage–current phase error	Non-inductive reference load	$R_{s,\mathrm{HV}}$, $X_{s,\mathrm{HV}}$, $G_{\mathrm{HV}}$, $E_{\mathrm{burst}}$	B	0.30° for 10–60kHz; ±0.50° over 8–100kHz
Hydrophone sensitivity	HBK 8103	$V_{h}$, pressure, TVR	B	$M_{h}\left(f\right)$; nominal −211dBre1V/μPa; u=±1.0dB
Source–receiver distance	Manual positioning	TVR	B	r=1.00m; u(r)=0.01m; $u_{r}\left(r\right)=1.0\%$
Impedance-analyzer accuracy	Tonghui TH2840B	Small-signal |Z|, phase, $R_{s}$, $X_{s}$	B	Basic accuracy 0.05%; frequency accuracy 0.01%
Repeatability	Five repeated bursts	All extracted parameters	A	N=5; representative RSD values of approximately 2.0% for electrical amplitude parameters, 4.0% for $E_{\mathrm{burst}}$, and 0.5dB for TVR
Adaptive-window selection	Threshold/window sensitivity analysis	$V_{1}$, $I_{1}$, *Z*, $E_{\mathrm{burst}}$	B	0.5% for $V_{1}$, 0.8% for $I_{1}$, 1.5% for *Z*, and 1.8% for $E_{\mathrm{burst}}$
Reinstallation reproducibility	Reinstallation/different-day test	$V_{h}$, TVR, $E_{h}$	A	Approximately 5–9% for hydrophone-related amplitude/energy indicators and up to 1.23dB for TVR

**Table 4:** Estimated combined uncertainties of the extracted parameters.

Parameter	$u_{c}$	*U* (k=2)
$V_{1,\mathrm{rms}}$	2.3%	4.6%
$I_{1,\mathrm{rms}}$	2.4%	4.8%
$|Z_{\mathrm{HV}}|$	3.3%	6.6%
$R_{s,\mathrm{HV}}$, $X_{s,\mathrm{HV}}$	3.8%	7.6%
$E_{\mathrm{burst}}$	4.5%	9.0%
$V_{h,\mathrm{pp}}$	7.7%	15.4%
$E_{h}$	8.5%	17.0%
$A_{h}\left(f_{\mathrm{exc}}\right)$	7.7%	15.4%
Effective short-burst TVR	1.3 dB	2.6 dB

**Table 5:** Correlation coefficients between electrical indicators and hydrophone response indicators. Each cell gives Pearson *r*/Spearman ρ.

Electrical Indicator	Hydrophone Response Indicator		
	$V_{h,\mathrm{pp}}$	$E_{h}$	$A_{h}\left(f_{\mathrm{exc}}\right)$
*E*	0.696/0.772	0.676/0.824	0.655/0.777
$I_{1}$	0.423/0.516	0.409/0.583	0.447/0.624
$R_{s,\mathrm{HV}}$	0.533/0.465	0.227/0.388	0.098/0.310

**Table 6:** Electrical characterization and natural-rock model-well results for eight nominally identical transmitting transducers.

Tx	$f_{r}$	$f_{a}$	$|Z_{\mathrm{SS}}|$	$\theta_{\mathrm{SS}}$	$G_{\mathrm{SS}}$	$V_{\mathrm{rms}}$	$I_{\mathrm{rms}}$	$|Z_{\mathrm{HV}}|$	$\theta_{\mathrm{HV}}$	$G_{\mathrm{HV}}$	$E_{\mathrm{burst}}$	$A_{P}$	$E_{P}$
	(kHz)	(kHz)	(Ω)	(°)	(mS)	(V)	(A)	(Ω)	(°)	(mS)	(J)	(V)	(V^2^ms)
T01	17.6	19.4	37.32	−14	26.0	355	9.52	37.30	−15.8	25.8	0.446	0.570	0.470
T02	17.1	19.0	42.00	−19	22.5	348	10.27	33.88	−13.5	28.7	0.536	0.924	1.009
T03	18.2	19.8	35.31	−11	27.8	360	10.48	34.34	−12.8	28.4	0.498	1.025	1.024
T04	17.4	19.1	38.42	−16	25.0	342	11.50	29.73	−8.1	33.3	0.576	1.642	1.881
T05	18.5	20.3	46.19	−24	19.8	365	8.52	42.82	−18.1	22.2	0.467	0.547	0.506
T06	17.0	19.0	34.66	−9	28.5	350	11.31	30.95	−9.1	31.9	0.564	1.520	1.696
T07	18.0	19.7	40.29	−17	23.8	352	10.57	33.29	−12.7	29.3	0.546	0.941	1.005
T08	17.8	19.7	39.80	−13	24.5	340	14.45	23.52	−5.7	42.3	0.649	1.700	1.970

## Data Availability

The data presented in this study are available from the corresponding author upon reasonable request.
